# Shoulder Bone Segmentation with DeepLab and U-Net

**DOI:** 10.3390/osteology4020008

**Published:** 2024-06-11

**Authors:** Michael Carl, Kaustubh Lall, Darren Pai, Eric Chang, Sheronda Statum, Anja Brau, Christine B. Chung, Maggie Fung, Won C. Bae

**Affiliations:** 1 General Electric Healthcare, Menlo Park, CA; 2 Dept. of Electrical and Computer Engineering, University of California-San Diego, CA; 3 Canyon Crest Academy, San Diego, CA; 4 Dept. of Radiology, VA San Diego Healthcare System, San Diego, CA; 5 Dept. of Radiology, University of California-San Diego, La Jolla, CA

**Keywords:** glenohumeral, glenoid, humeral head, image processing, ZTE, MRI, U-Net, DeepLab

## Abstract

Evaluation of 3D bone morphology of the glenohumeral joint is necessary for pre-surgical planning. Zero echo time (ZTE) magnetic resonance imaging (MRI) provides excellent bone contrast and can potentially be used in place of computed tomography. Segmentation of shoulder anatomy, particularly humeral head and acetabulum, is needed for detailed assessment of each anatomy and for pre-surgical preparation. In this study we compared performance of two popular deep learning models based on Google’s DeepLab and U-Net to perform automated segmentation on ZTE MRI of human shoulders. Axial ZTE images of normal shoulders (n=31) acquired at 3-Tesla were annotated for training with a DeepLab and 2D U-Net, and the trained model was validated with testing data (n=13). While both models showed visually satisfactory results for segmenting the humeral bone, U-Net slightly over-estimated while DeepLab under-estimated the segmented area compared to the ground truth. Testing accuracy quantified by Dice score was significantly higher (p<0.05) for U-Net (88%) than DeepLab (81%) for the humeral segmentation. We have also implemented the U-Net model onto an MRI console for a push-button DL segmentation processing. Although this is an early work with limitations, our approach has the potential to improve shoulder MR evaluation hindered by manual post-processing and may provide clinical benefit for quickly visualizing bones of the glenohumeral joint.

## 1. Introduction

Evaluation of patient-specific 3D position and morphology of the glenohumeral joint bone is useful clinically for multitude of reasons. These include a basic diagnosis of shoulder dislocation and fracture, visualization of glenoid surface for determining glenoid bone loss and fracture [1,2], and measurement of bone morphology for pre-surgical planning,[3–6] needed for shoulder arthroplasty [7].

**Figure 1.**
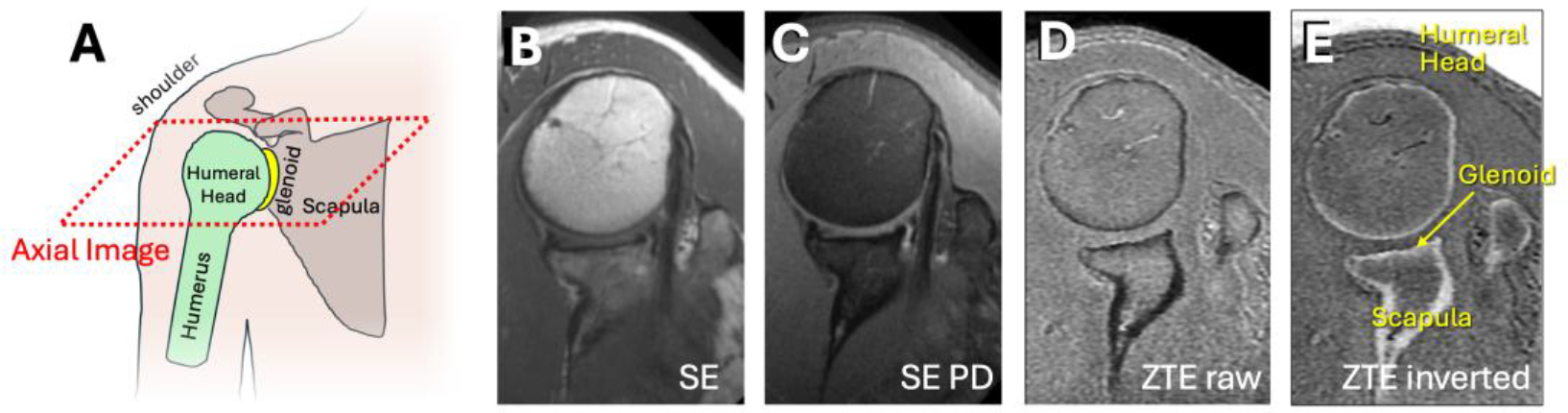
(A) Anatomy of shoulder showing major bone structures of humerus, humeral head, glenoid, scapula. Axial imaging plane is shown in dotted red box. Conventional shoulder MR images acquired using conventional (B) spin echo proton density weighted, (C) spin echo proton density weighted with fat suppression, and (D) zero echo time (ZTE) sequences. Conventional sequences do not isolate bone effectively. (E) Inverted ZTE image depicting mostly bony tissues with high signal intensity. Conventional MR images depict non-bony tissues with similar contrast as bony tissues, making them less useful for bone-only imaging.

Computed tomography (CT) is the current gold standard for bone imaging in 3D [6,8–10]. While preferred for the lack of ionizing radiation, conventional magnetic resonance imaging (MRI) sequences (Figure 1AB, provided as examples) have suboptimal contrast (i.e., bone has similar signal as several other tissues) for distinguishing bone for visualization [9]. However, recent advances in MRI including ultrashort time-to-echo (UTE) [11,12] and zero time-to-echo (ZTE) techniques [13–16] have shown to be promising alternatives that can depict cortical bone with a uniform contrast (i.e., low signal for the cortical bone and the air/back ground, high signal for all other tissues such as muscle and trabecular bone and marrow; Figure 1C), making it relatively easy to isolate and visualize the bones [14,17], with minor processing. Unlike conventional MR images that depict cortical bones and other soft tissues (such as ligaments) with low signal intensity (Figure 1AB), ZTE MRI provides a more uniform contrast for bone, for example with a high signal intensity in inverted ZTE images (Figure 1D). These studies have also shown that cortical bone morphology (e.g., surface contour) measured on UTE or ZTE are highly similar to that measured on a CT or a micro CT [17]. For these reasons, ZTE MRI is increasingly being prescribed when bone imaging is desired [17].

As mentioned above, it is useful to visualize and analyze individual bones of the glenohumeral joint (Figure 1A). This requires manual segmentation, a time-consuming process. This can be improved with traditional segmentation techniques utilizing thresholding of pixel values [18], region growing [19], active shape modeling [20]. Recently, deep learning techniques such as full convolutional network (FCN) [21] and U-Net [22] have been highly successful in performing image segmentation using small training data with good results. Another development includes Google’s DeepLab, designed to perform image segmentation [23]. This model consists of 3 main components of feature extraction module, an atrous spatial pyramid pooling (ASPP) module, and a decoder module. It has been used widely for scene segmentation and to a limited degree for medical imaging for tumors [24–26], but not for bone segmentation..

Specifically for segmentation of shoulder MRI, several recent studies have used deep learning methods. One study used [27] 2D and 3D U-Net to segment the humeral head and glenoid on conventional spin echo and gradient echo MR images. Another approach [27–29] combined U-Net and AlexNet to perform rough and fine segmentation, respectively, of the humeral head and the glenoid bones. Others also used convolutional network for shoulder bone segmentation [28–31], or bone recognition for shoulder muscle segmentation [32]. DeepLab, although readily available, has not been used previously for this application. It would be important to compare not only performance of several models, but also nuances of how different models behave.

Additionally, past studies focused solely on the performance of the model, not on the important aspect of how the segmentation integrates with the existing workflow. Vast majority of deep learning models require off-line processing for inference, and the raw output may not be compatible with dicom viewers or picture archiving and communication system (PACS) that maybe used by clinicians [33–35]. We aim to demonstrate an implementation that would be useful for clinical workflow.

The goal of this study was to demonstrate an end-to-end approach for creating and deploying deep learning-based models to perform shoulder MRI segmentation, comparing two common deep learning models, to provide immediate utility without the hurdle of off-line processing. This is the first study to compare deep-learning based shoulder segmentation using U-Net and DeepLab, and we also describe an implementation for achieving this directly on the console, which may facilitate a quick clinical translation.

## 2. Materials and Methods

Parts of this study involving MR imaging of human subjects was approval by the institutional review board. Remaining images were obtained after de-identification.

**Figure 2.**
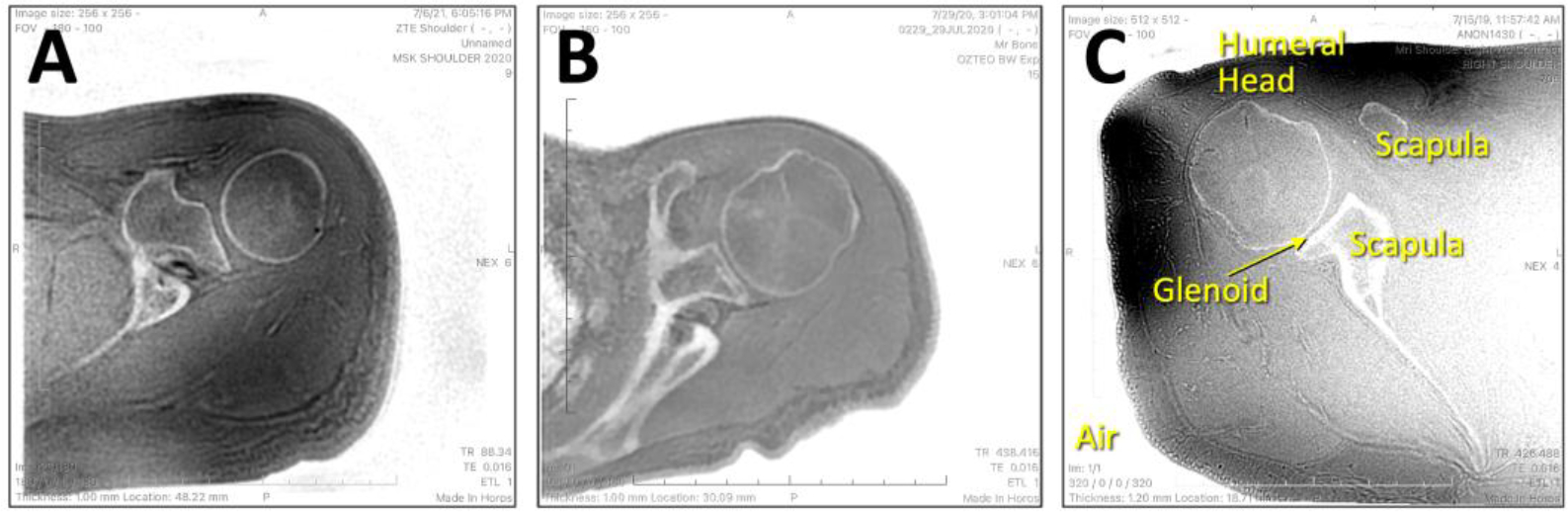
Inverted axial ZTE shoulder images used in this study were acquired with moderately varying scan parameters. (A) was acquired with TR=88 ms, TE=0.016 ms, FOV=180 mm, matrix=256×256, and 1 mm slice thickness. (B) was acquired with TR=458 ms, TE=0.016 ms, FOV=160 mm, matrix=256×256 and 1 mm slice thickness. (C) was similar to (B) but acquired with FOV=180 mm, matrix=512×512, and 1.2 mm slice thickness. While varying in image contrast, all images shared a similar feature of depicting bones of the shoulder with high signal intensity.

**Figure 3.**
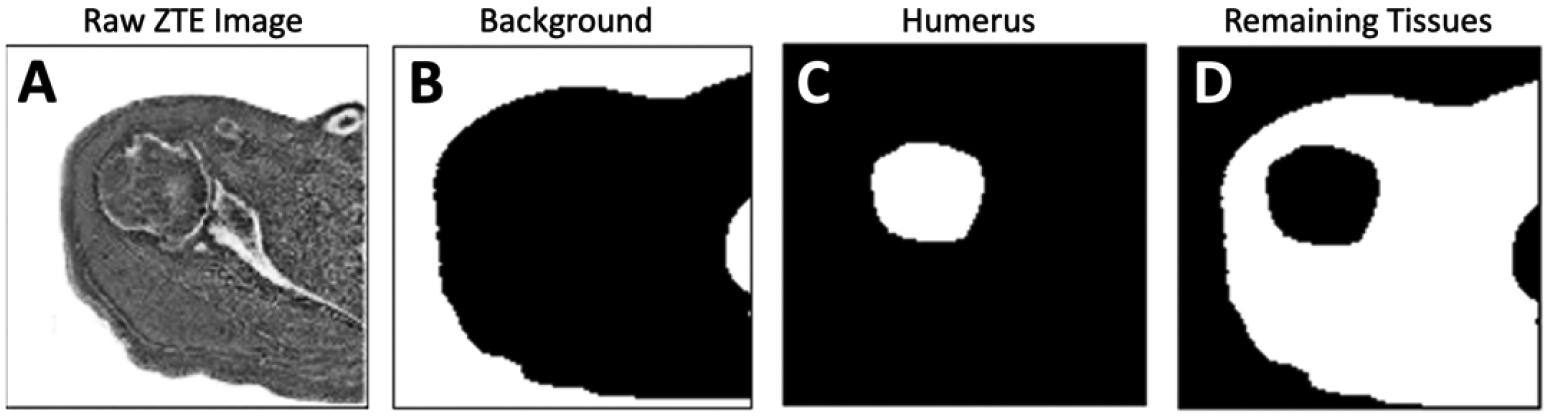
Manual segmentation of the MRI images. (A) Inverted ZTE MRI shoulder images acquired in the axial plane were manually annotated (segmented) into (B) background, (C) humeral head / humerus, and (D) the remaining tissues.

The overview of the methodology is as follows: (1) acquire or collect ZTE MRI images of the shoulder similar to Figure 2; (2) pre-process images for uniform size and grayscale values (see section 2.1. Pre-processing); (3) split data into training and testing sets; (4) perform annotation which is manual segmentation of the images into three regions as shown in Figure 3; (5) perform training separately for U-Net and DeepLab models; here, pre-processed images will be the inputs, and the segmented images will be the outputs to be compared against manual segmentation images; (6) after training, perform inference on test data and determine accuracy; (7) finally, implement a deep learning model on the MRI console for immediate processing.

### 2.1. Zero Echo Time (ZTE) MRI Data

#### Training Data:

For deep learning (DL) training data, we obtained de-identified MRI shoulder dataset through an existing study where 7,935 images from n=31 normal asymptomatic unilateral (left or right) shoulders, acquired on General Electric 3-Telsa scanners, were available. The images were acquired in the axial plane using ZTE sequence with mixed scan parameters: Time-to-Repetition (TR)=100 to 600 ms, Time-to-Echo (TE)=0.016 to 0.028 ms, field of view (FOV)=160 to 240 mm, image matrix=256×256 to 512×512, slice thickness=0.7 to 1.2 mm, number of slices=90 to 200. Despite some variations in the scan parameters, the images had generally similar appearances (Figure 2A to C), depicting bone and air with low signal intensity, the most of other soft tissues with a moderate to high signal intensity. As a part of standard processing on the MRI console, raw ZTE images were intensity-corrected, and then inverted (i.e., from Figure 1C to D) to depict bone with high signal intensity (Figure 2). Examples of inverted axial ZTE shoulder images are shown in Figures 1D and 2. Unfortunately, demographical information (age, sex, etc.) was not available.

#### Testing Data:

For testing data, additional axial ZTE shoulder data (1860 images) were obtained from n=13 subjects that included an existing data from nine volunteers (demographic information was not available) and four newly acquired data from subjects with recent shoulder pain (3 males, 1 female; age range 40 to 55 years old). These were all acquired with similar scan parameters as the training data.

#### Pre-Processing:

Training and testing data were pre-processed prior to inputting into the deep learning models by normalizing the voxel values in each 3D stack (separately for each shoulder) between 0 and 1, and conversion to 8-bit image with voxel values between 0 and 255. The images were then resized to 256×256 in-plane using bilinear interpolation.

### 2.2. Opportunistic CT Data for Comparison

We obtained an opportunistic data, where both a clinical CT scan and a ZTE MRI of the same shoulder was available. ZTE scan parameters were similar to the training data. CT scanning parameters were: voltage=140 kVp, current=120 mA, reconstruction diameter=275 mm, image matrix=512×512, and slice thickness=1.25mm. The images data were first registered using Matlab, then the registered ZTE data was segmented using DL, while the CT data was segmented manually. Dice score for humerus segmentation was determined, and the respective segmented images were fused, and 3D rendered for visual comparison.

### 2.3. Annotation / Manual Segmentation

All images were annotated using ImageJ [36] (v2.1.0). Inverted ZTE MR images (Figure 3A) were loaded as a three-dimensional (3D) stack and annotated using Segmentation Editor plugin. On every 2 to 3 slices, boundaries of the humerus was drawn using polygon selection tool, interpolated between slices, then filled to create a binary 3D image for the humerus (Figure 3C). For the background segmentation (Figure 3B) we performed thresholding and additional manual clean-up. Finally, segmentation for the remaining tissues was created by inversion of background image and subtraction of the humerus image. This yielded three separate binary 3D images, representing (i) the background / air (Figure 3B) including the bulk of the lung, (ii) humeral head and humerus bone (Figure 3C), and (iii) all other soft and bony tissues (i.e., glenoid, acromion, etc., Figure 3D). Note that humerus segmentation was performed loosely around the structure, to avoid accidental cropping of humeral head. While we used a single observer (a non-physician with 10 years of experience in imaging research, trained in musculoskeletal section), we felt that bone segmentation task did not require extensive training due to a high contrast between bone vs. soft tissues, and that our approach of using loosely fitting annotation allowed for rapid annotation that still included the structure of interest without error.

### 2.4. Deep Learning Segmentation Models

We have implemented two-dimensional (2D) U-Net [22] and DeepLab v3 [23] deep learning (DL) models in Matlab with Deep Learning Toolbox (R2021b) to perform the segmentation of shoulder ZTE MR images. The DL models first takes in inverted ZTE shoulder image that has been resized to 256×256 voxels.

**Figure 4.**
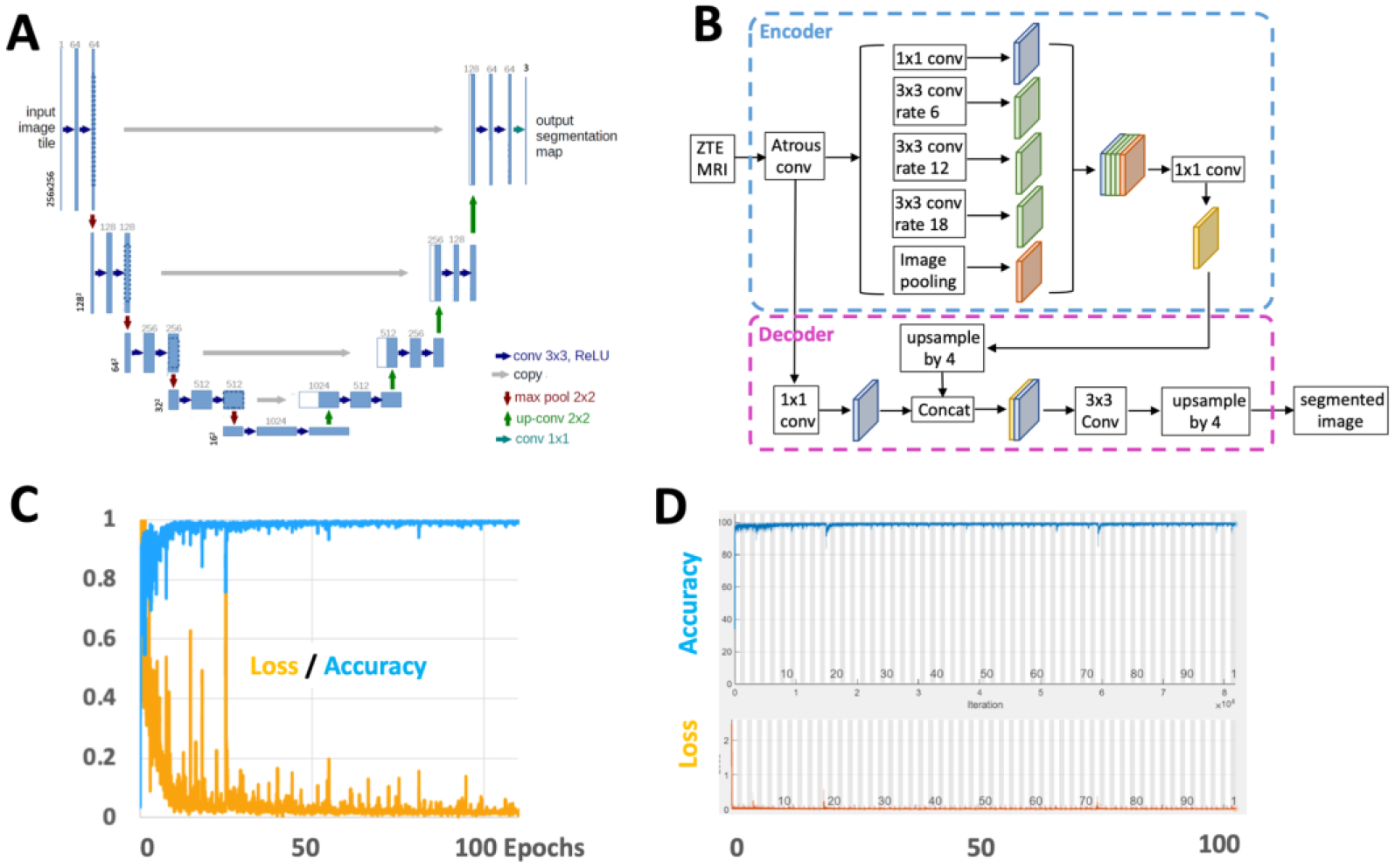
Architectures of (A) U-Net and (B) DeepLab used in this study. Adapted from [22] and [37], respectively. Training results showing accuracy and loss values for (C) U-Net and (D) DeepLab.

The U-Net (Figure 4A) applies 64 convolution filters with 3×3 kernel size, two convolution operations at each step, with an encoder depth of 5 (or 9 layers, 4 down-sampling followed by 4 up-sampling). The DeepLab v3 (Figure 4B) uses spatial pyramid pooling module for encoding, which captures contextual information at multiple scales using 3×3 convolution filters with different dilation rates of 6, 12, and 18. This is then pooled and concatenated in the decoder to produce the final segmentation.

In both models, the final output consists of pixel classification layer with 3 classes, each for the background (air), humeral head/humerus, and the remaining other tissues. The models were trained to 120 epochs (~2 days) using the default setting (Adam optimizer, L2 regularization, population batch normalization, shuffling image every epoch, mini batch size of 8) using cross entropy as the loss function. on a Windows 10 PC with i7-10700K CPU, 32GB RAM, RTX3090 GPU with 24GB VRAM. Training accuracy quickly converged to >99% (Figure 4C and 4D) and remained high. The weight that provided the lowest loss during training was kept and used for the remainder of the study. A Matlab code is provided below in 2.4.1. to clarify the model building and training processes. (We also trained on augmented images using rotation, translation, and rescaling, but unfortunately this did not improve the test results. Additionally, the current approach did not use cross-validation, which is a moderate limitation.)

#### 2.4.1. Matlab Code


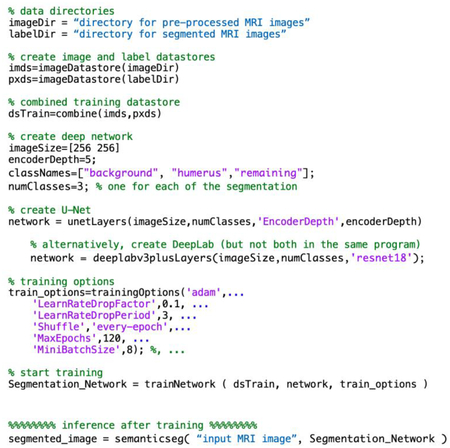


### 2.5. Inference Accuracy

After training the U-net model, DL segmentation was performed on the validation images (each dataset taking ~2 min) and compared against the manual annotation. Following similarity metrics were determined. Dice score [38,39] provides a measure of image (i.e., segmentation mask) overlap, defined as Eqn. 1:

(Eqn. 1) $$\text{Dice}=\left(2\;\text{TP}\right)/\left(2\;\text{TP}+\text{FP}+\text{FN}\right)$$

where TP is the number of true positive voxels (i.e., value of 1 in both DL and manual segmentations), FP is the number of false positive voxels (value of 1 in DL, value of 0 in manual segmentation), and FN is the number of false negative voxels (value of 0 in DL, value of 1 in manual segmentation).

Sensitivity [40] was determined as Eqn. 2:

(Eqn. 2) $$\text{Sensitivity}=\text{TP}/\left(\text{TP}+\text{FN}\right)$$

Specificity [40] was determined as Eqn. 3:

(Eqn. 3) $$\text{Specificity}=\text{TN}/\left(\text{TN}+\text{FP}\right)$$

where TN is the number of true negative voxels (value of 0 in both DL and manual segmentations).

### 2.6. Clinically Applicable DL Segmentation Workflow

**Figure 5.**
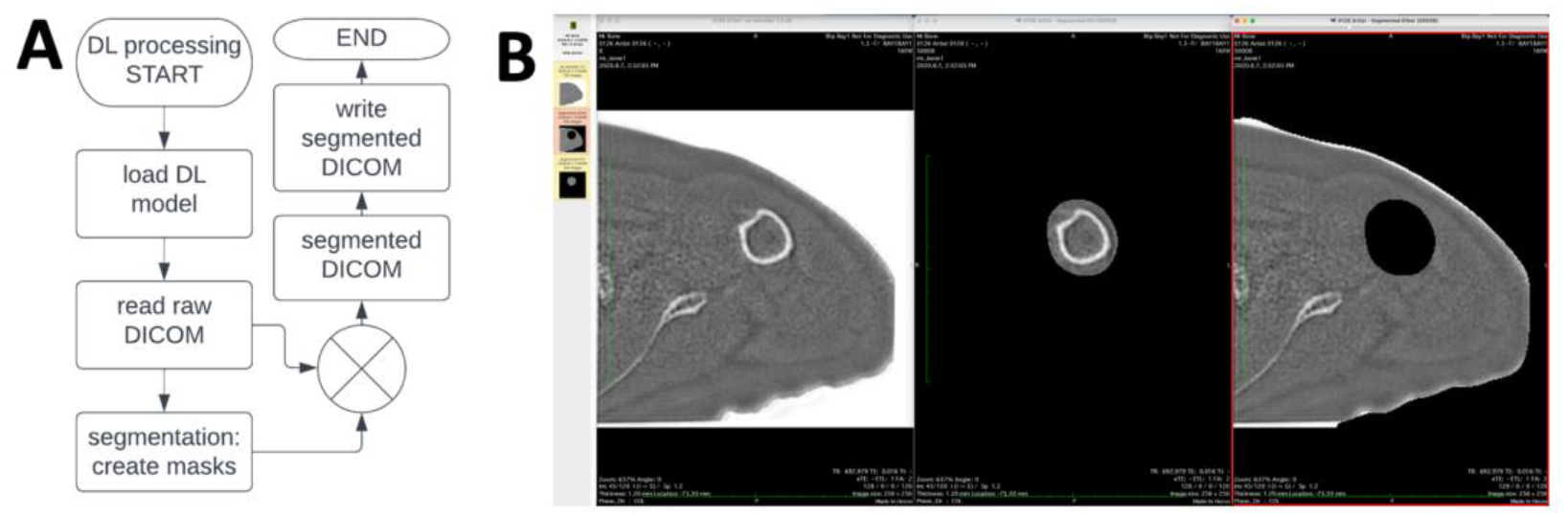
(A) Flow chart of ZTE DL processing, which reads raw DICOM images, performs DL segmentation to create masks for humerus and the remaining tissues. The masks are then multiplied with the raw image to create segmented DICOM images that are saved as new series in the exam. (B) Segmented DICOM images viewed in a PACS viewer, showing the original image on the left, segmented humeral bone in the middle, and segmented remaining tissues on the right.

In addition to the development of DL segmentation model, additional processing steps were developed to create a workflow that can be used on an MRI scanner, without the need for off-line processing. As shown in a flowchart in Figure 5A, a Matlab runtime (R2021b for Linux) program was compiled as binary and installed on a MRI console to: (1) read inverted ZTE dicom images, (2) perform DL segmentation (using the same routine as the off-line version) to create masks of humeral head/humerus and the remaining tissues, (3) multiply with input ZTE images to created new series of images showing the separated structures viewable on the MRI console or any PACS (Figure 5B). This enabled a push-button DL segmentation of ZTE shoulder images directly on the MRI scanner for an immediate evaluation following acquisition. As a quality control measure, we compared the dicom images from MRI console to the those from off-line programs and found no difference in the images.

### 2.7. Statistics

For the validation data, Dice scores, sensitivity, and specificity for segmentation of the humerus and the remaining tissues were tabulated and the mean and standard deviation of the values were determined and plotted as box plots. To determine the effect of different models in the segmentation performance, we compared the mean values between U-Net and DeepLab models using t-test with significance level set at 0.05, using Systat (v10, Grafiti LLC, Palo Alto, CA, USA).

## 3. Results

### 3.1. Training

Training results were good, converging to >99% accuracy (Figure 4C and 4D). The model weight that achieved the highest accuracy was kept and used for the remainder of the study.

### 3.2. Testing

**Figure 6.**
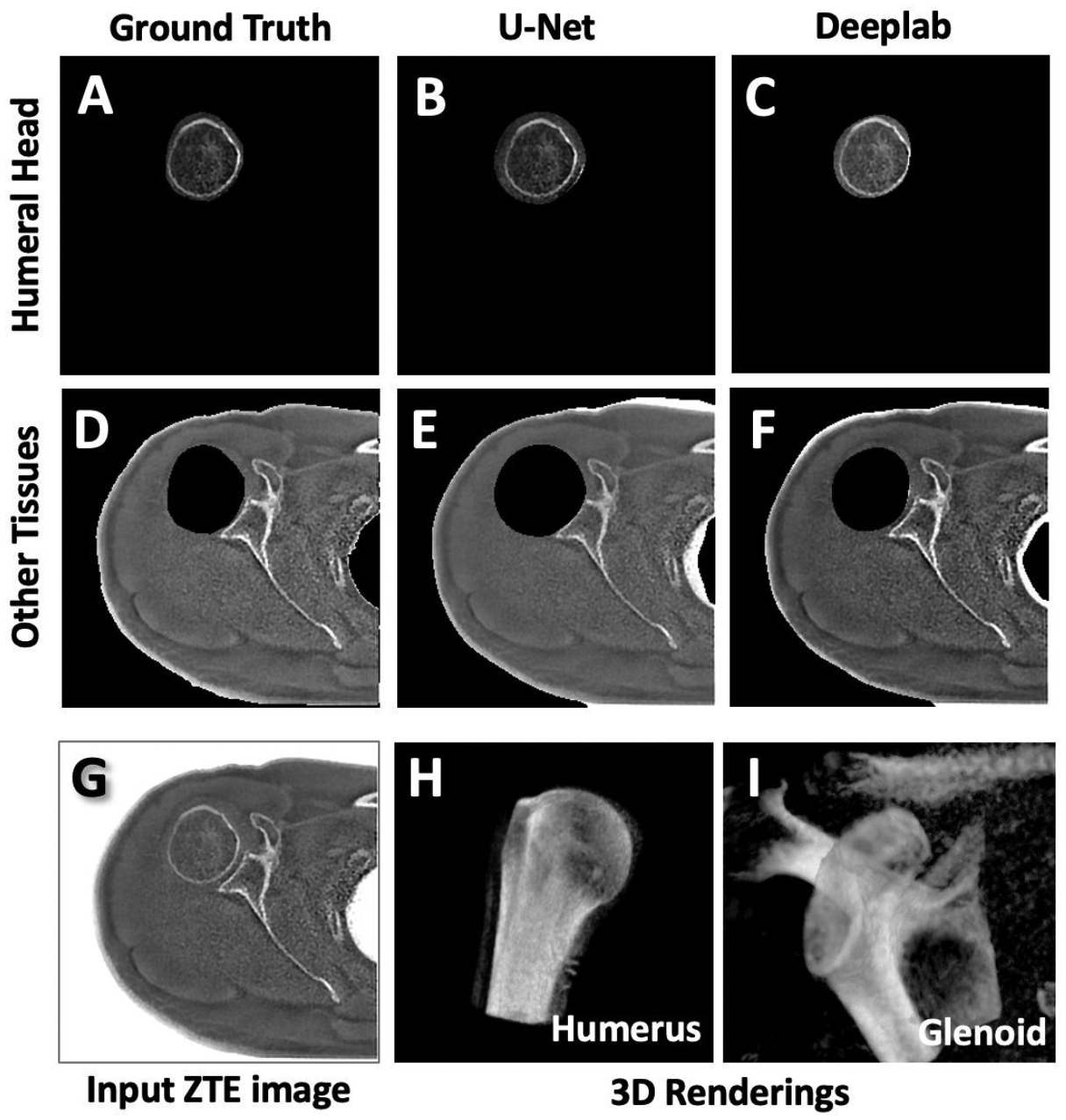
Segmentation results on test images. (A, D) Ground truth or manually segmented images of humeral bone and the remaining other tissues shown for comparison. Output segmented images of (B, C) the humeral head (E, F) and the remaining tissues after DL segmentation performed by (B,E) U-Net and (C,F) DeepLab. Qualitatively, U-Net slightly over-estimated area for humeral head while DeepLab slightly under-estimated. (G) Input ZTE MRI image is shown. (H, I) Segmented ZTE images (from the ground truth; A and D) were used to create separate 3D renderings of the (H) humerus and (I) glenoid / scapular bone.

Figure 6A to F shows segmented ZTE test images. Ground truth segmentations (Figure 6AD) are shown next to DL segmentation performed by U-Net (Figure 6BE) and DeepLab (Figure 6CF). While both models performed well by including the important areas (i.e., bone) in the humeral head segmentation, U-net slightly over-estimated segmentation for humeral head while DeepLab slightly under-estimated. The segmented ZTE images could be used for 3D morphologic evaluation of the humeral head (Figure 6H) and the glenoid (Figure 6I).

**Figure 7.**
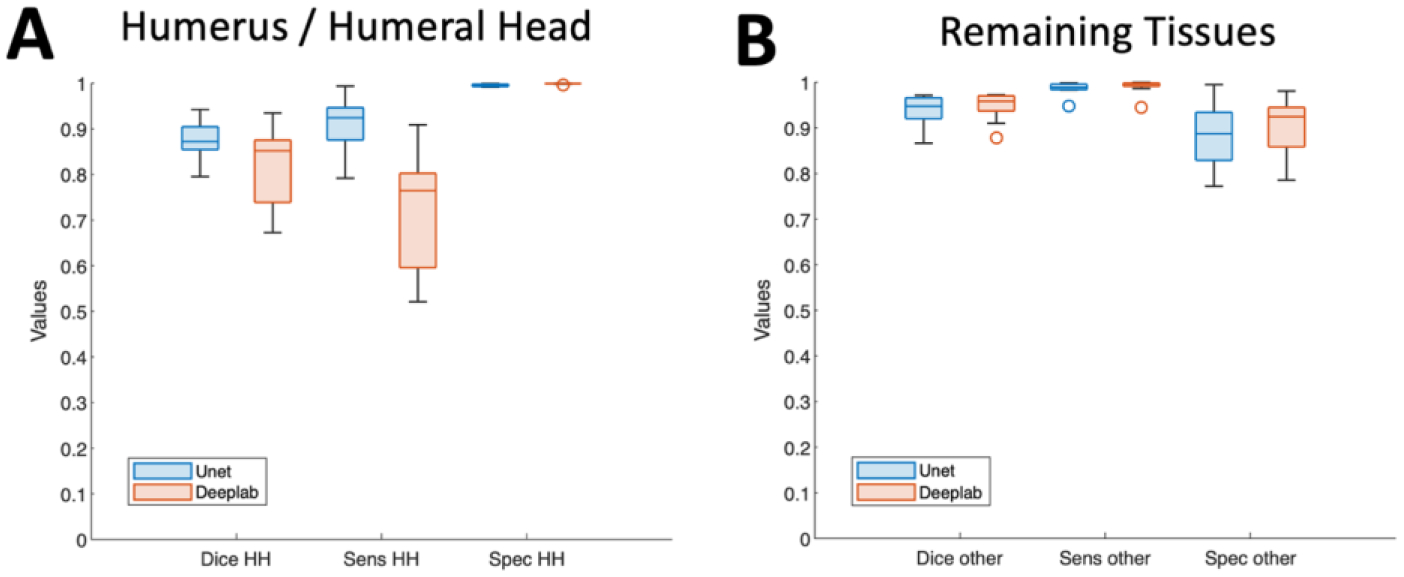
DL model performances compared. Boxplots of inference accuracy (Dice score, sensitivity, specificity) quantified on the humeral bone (A) and the remaining tissue (B), determined using U-Net (blue) and DeepLab (red) models. Marked differences in the accuracy metrics for the humeral bone was noted.

Figure 7 shows testing results quantified using Dice score, sensitivity, and specificity. Figure 7A is the boxplot for humerus/humeral head and Figure 7B is the boxplot for the remaining other tissues, and blue color represents U-Net and red color represents DeepLab.

Table 1 shows the mean and standard deviation, and p-value suggesting the difference in values between the models. U-Net had consistently high Dice scores throughout, averaging 0.88 for humerus/humeral head and 0.94 for the remaining tissues. Sensitivity and specificity values were also very high, ranging from 0.88 to 0.99. In comparison, DeepLab had significantly lower Dice score (0.81 vs. 0.88, p=0.027) and sensitivity (0.71 vs. 0.91, p<0.001), but greater specificity (0.999 vs 0.995, p<0.001), for the humerus/humeral head segmentation. Other metrics were not statistically different. This, combined with visual comparison, suggested that U-Net was generally more sensitive and slightly over-included areas of humeral head, while DeepLab was more conservative in this regard.

### 3.3. Comparison vs. CT

**Figure 8.**
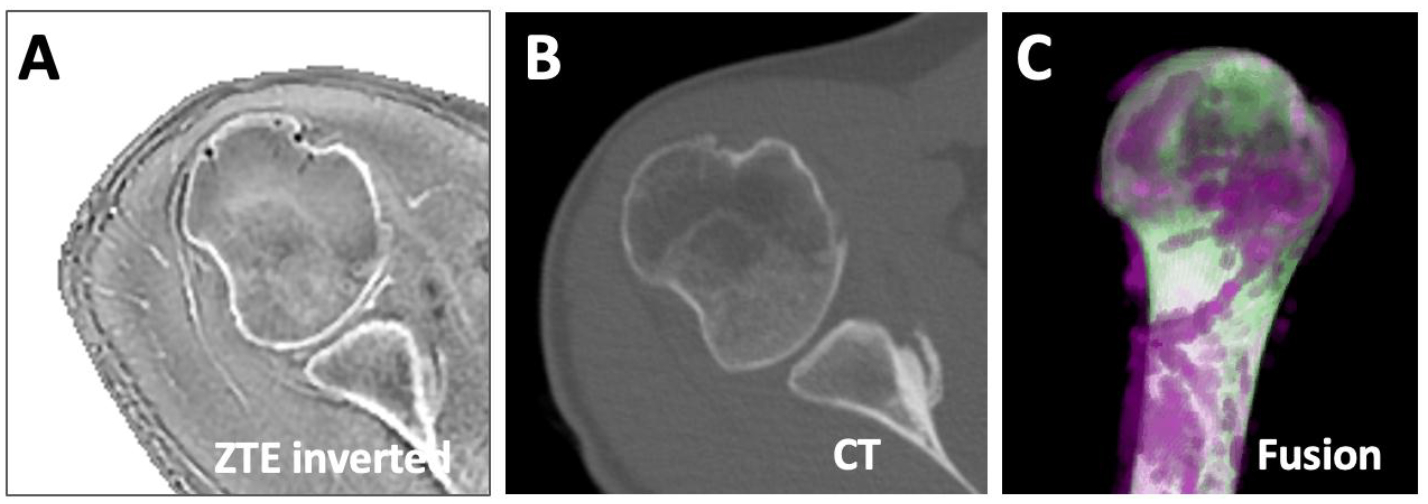
Comparison of MRI vs. CT segmentation. ZTE MRI (A) and CT (B) data of the same subject were registered and segmented (using U-Net for MRI, manually for CT). The segmented images were fused (C), showing the overlapping regions as white, and the non-overlapping regions in magenta for MRI and green for CT.

In the opportunistic data where both ZTE MRI (Figure 8A) and CT (Figure 8B) data were available from the same subject, the Dice score of humerus segmentation using DL was 97%. We created a fused 3D rendering (Figure 8C), which shows an excellent overlap (white) between ZTE (purple) and CT (green), and likely to yield similar values when measured for length, etc. However, this needs to be validated in additional samples.

## 4. Discussion

We have successfully implemented DL model to segment the humerus and other tissues from ZTE MRI shoulder images. Using a small number of training data, a reasonable level of performance was achieved both visually and quantitatively with moderately high Dice scores, sensitivity and specificity using both U-Net and DeepLab models. Comparison of segmented ZTE vs. CT showed an excellent overlap, suggesting that ZTE MRI could become useful modality for imaging bony tissues in the body. The DL model has also been implemented on a MRI scanner to perform a push-button automated segmentation in 2 to 3 minutes. This work could be useful clinically when it is desired to evaluate 3D bone morphology at the glenohumeral joint for bone defects [41] or dysplasia [42], or for pre-surgical measurement [3–6], by eliminating the need for manually segmenting the humeral bone from the glenoid.

While there have been a few studies on DL segmentation of shoulder MRI, this is the first study utilizing ZTE images, and using DeepLab architecture. Compared to other DL segmentation models trained on conventional MRI, performance of our model may appear underwhelming at first. Rodrigues et al. [27] used a 2D U-Net model to achieve sensitivity and specificity of 95%, and 99%, respectively, for the humerus, and 86% and 92%, respectively, for the glenoid. Mu et al. [29] and Wang et al. [28] both achieved average sensitivity of ~95% for segmenting the two bones. One study utilizing a conventional thresholding method combined with a manual selection [43] found that MRI measure of glenoid area correlated strongly (intraclass correlation coefficient of 0.94) with that from CT. In contrast, our models achieved a somewhat lower average sensitivity of 71% (DeepLab) and 92% (U-Net) but a high specificity of >99% (both models) for the humerus segmentation. This may be related to our approach of using a loosely oversized region (Figure 6A) for annotating the humerus bone, unlike in past studies that needed to use precisely defined region for each bone [27]. While our approach may have resulted in a lowered sensitivity, minor deviations in the segmentation mask do not appear to cause problems for the simple purpose of isolating the humeral head and glenoid for visualization. For additional quantifications such as volume measurement, conventional image processing techniques (e.g., thresholding [18,44–46]) may be applied to further segment the bone from surrounding soft tissues.

Comparison between U-Net vs. DeepLab suggested that while both yielded visually satisfactory results, there was tendency of U-Net to slightly over-estimate the area for the humerus segmentation, while DeepLab tended to slightly under-estimate. A consequence of this can be seen in Figure 6, where U-Net segmentation included a sliver of glenoid bone (Figure 6B), while DeepLab missed a sliver of humeral bone (Figure 6C). This was also apparent in a significantly higher sensitivity of humeral segmentation for U-Net, and a significantly higher specificity of humeral segmentation for DeepLab. Although exact reasons for such differences are unclear, DeepLab’s architecture that uses atrous (i.e., dilated) convolutions tends to broaden the receptive field, which maybe advantageous for detection and inclusion, but may make the segmentation less precise.

This early study has several limitations. First, we used only axial images for the training and testing. Given the 2D nature of the models, shoulder images in other planes (coronal, sagittal) will not provide the expected results in the present study. However, ZTE images are often acquired as an isotropic 3D stack [16], so axial reformatting will not degrade the image quality. The variations in scan parameters of the training images, while lowering segmentation accuracy, would have been beneficial in terms of generalizability of the model. Segmentation was performed by a single observer, which is less desirable than using an average of multiple observers. However, this may not be critical as we utilized loosely fitting masks unlike most other studies. U-Net and DeepLab models have been introduced some time ago and there are newer models such as Segment Anything Model. However, the application of the existing models to the shoulder segmentation and the comparison of the results still provide useful insight. Although our study did not specifically try transfer learning [30,47] from existing weights and models. Additionally, U-Net and DeepLab models are readily available on Matlab platform for easier adaptation, unlike the newest models that may require expertise in computer science discipline. The number of training datasets (7,935 images from 31 subjects), while sufficient to yield a functional model, was too small to capture large variations found in normal shoulder anatomy and did not include any shoulders with known abnormalities in bone morphology [48–51]. Additional and varied datasets in the future will likely improve segmentation performance. ZTE MRI, while providing superior bone contrast compared to conventional MR sequences, still falls short compared to CT scan for bone evaluation. Tissues other than bone can appear iso-intense with bone (Figure 8A) and this may adversely affect visualization (Figure 8C, purple signal) using our loosely encompassing segmentation masks.

## 5. Conclusions

In conclusion, we developed and deployed a fully automated methodology based on two popular deep learning models to segment humerus and other bones on novel ZTE MR images of the human shoulder. Although this is an early attempt with limitations, with additional training data and model refinement, this study has a potential to improve clinical practice, by improving clinical workflow such as evaluation of 3D bone morphology or pre-surgical measurement of the glenohumeral joint, by providing rapid and automated segmentation of the humeral bone.

## Figures and Tables

**Table 1.** Mean and standard deviation of the Dice scores, sensitivity, and specificity values. P-values from t-tests indicate statistical difference between the mean values obtained using U-Net vs. DeepLab.

		Dice HH	Dice other	Sens HH	Sens other	Spec HH	Spec Other
**U-Net**	Mean	**0.876**	**0.940**	**0.910**	**0.987**	**0.995**	**0.883**
	SD	0.043	0.031	0.061	0.013	0.003	0.070
	N	13	13	13	13	13	13
**DeepLab**	Mean	**0.811**	**0.949**	**0.715**	**0.992**	**0.999**	**0.903**
	SD	0.088	0.030	0.132	0.015	0.001	0.055
	N	13	13	13	13	13	13
**p-value**		**0.027**	0.467	**<0.001**	0.426	**<0.001**	0.424

## Data Availability

The data are not publicly available due to privacy issues.
